# Gafchromic EBT3 film dosimetry in electron beams — energy dependence and improved film read‐out

**DOI:** 10.1120/jacmp.v17i1.5970

**Published:** 2016-01-08

**Authors:** Petri Sipilä, Jarkko Ojala, Sampsa Kaijaluoto, Ilkka Jokelainen, Antti Kosunen

**Affiliations:** ^1^ Radiation Practices Regulation, Radiotherapy and Nuclear Medicine STUK—Radiation and Nuclear Safety Authority Helsinki Finland; ^2^ Department of Oncology Unit of Radiotherapy, Tampere University Hospital Tampere Finland; ^3^ Department of Medical Physics Medical Imaging Center, Tampere University Hospital Tampere Finland; ^4^ Radiation Practices Regulation, Radiation Metrology Laboratory and Occupational Exposure STUK—Radiation and Nuclear Safety Authority Helsinki Finland

**Keywords:** dosimetry, radiochromic film, Gafchromic, EBT3, Monte Carlo

## Abstract

For megavoltage photon radiation, the fundamental dosimetry characteristics of Gafchromic EBT3 film were determined in ${\;}^{60}\text{Co}$ gamma ray beam with addition of experimental and Monte Carlo (MC)‐simulated energy dependence of the film for 6 MV photon beam and 6 MeV, 9 MeV, 12 MeV, and 16 MeV electron beams in water phantom. For the film read‐out, two phase correction of scanner sensitivity was applied: a matrix correction for scanning area and dose‐dependent correction by iterative procedure. With these corrections, the uniformity of response can be improved to be within ±50 pixel values (PVs). To improve the read‐out accuracy, a procedure with flipped film orientations was established. With the method, scanner uniformity can be improved further and dust particles, scratches and/or dirt on scanner glass can be detected and eliminated. Responses from red and green channels were averaged for read‐out, which decreased the effect of noise present in values from separate channels. Since the signal level with the blue channel is considerably lower than with other channels, the signal variation due to different perturbation effects increases the noise level so that the blue channel is not recommended to be used for dose determination. However, the blue channel can be used for the detection of emulsion thickness variations for film quality evaluations with unexposed films. With electron beams ranging from 6 MeV to 16 MeV and at reference measurement conditions in water, the energy dependence of the EBT3 film is uniform within 0.5%, with uncertainties close to 1.6% (k=2). Including 6 MV photon beam and the electron beams mentioned, the energy dependence is within 1.1%. No notable differences were found between the experimental and MC‐simulated responses, indicating negligible change in intrinsic energy dependence of the EBT3 film for 6 MV photon beam and 6 MeV–16 MeV electron beams. Based on the dosimetric characteristics of the EBT3 film, the read‐out procedure established, the nearly uniform energy dependence found and the estimated uncertainties, the EBT3 film was concluded to be a suitable 2D dosimeter for measuring electron or mixed photon/electron dose distributions in water phantom. Uncertainties of 3.7% (k=2) for absolute and 2.3% (k=2) for relative dose were estimated.

PACS numbers: 87.53.Bn, 87.55.K‐, 87.55.Qr

## I. INTRODUCTION

Electron beams have been the primary treatment modality for skin and superficial malignancies throughout the era of modern radiotherapy. Owing to the physical properties (i.e., high dose to the surface and first centimeters below the skin surface in the regions of treatment volumes and sharp dose falloff at depths larger than the depth of dose maximum ($d_{\text{max}}$) to spare organs at risk), electron beam radiotherapy has stood up against increasing popularity of photon beam radiotherapy, which has advanced along with new treatment techniques such as intensity‐modulated radiotherapy (IMRT) and volumetric‐modulated arc radiotherapy (VMAT).^(1)^ In addition to recent advancements in electron beam radiotherapy (e.g., dynamic electron arc therapy^(2)^), there are also cases where a combination of an electron beam and photon IMRT fields or VMAT arcs could produce a better dose distribution for the patient than one beam quality solely.^1^, ^3^, ^4^, ^5^

The accuracy of dosimetry of electron and combined electron/photon beam dose distributions in phantom is challenged, mainly due to required high spatial resolution in areas of large dose gradients involved. Moreover, the gradients of electron spectra in phantom and measurements in combined photon/electron fields emphasize the low energy dependence of the dosimeter dose response. To fulfill the need to measure two‐dimensional dose distributions, radiochromic films have successfully been applied in external and internal radiotherapy for kilovoltage to megavoltage beam energies with various beam types, including photon, electron, and proton beams.^(6)^

Gafchromic EBT films (Ashland ISP, Wayne, NJ) are the most popular radiochromic films and, at the moment, the third generation of the film (EBT3) is available for the radiotherapy community. Several studies have been published presenting the dosimetric characteristics of the EBT3 film, often comparing to the previous EBT2 model.^7^, ^8^, ^9^, ^10^, ^11^, ^12^, ^13^ Many groups have also published dosimetry protocols or comprehensive sets of dosimetry procedures based on the use of the EBT3 film.^14^, ^15^, ^16^, ^17^, ^18^, ^19^, ^20^ It has been widely used for various applications, for example verification of IMRT treatment plans,^(21)^ stereotactic radiotherapy,^22^, ^23^ *in vivo* dosimetry,^(24)^ and brachytherapy.^(25)^ However, the number of reported results on dosimetric characteristics of the EBT3 film in electron beam dosimetry is limited and the studies applying the EBT3 film to various purposes in electron beam radiotherapy are almost nonexistent. Even though some studies suggest that the performance of the EBT3 film would be comparable to its predecessor, EBT2 film,^7^, ^13^ it is also known that the structure and/or the atomic composition of various used materials between different EBT film generations have varied, and even during the product life span.^11^, ^26^ Therefore, it is important to evaluate the performance of every new EBT film model and always, when changes in the structure and/or atomic composition are made, with every beam quality. Sorriaux et al.^(10)^ studied the characteristics of the EBT3 film dosimetry system in clinical photon, proton, and electron beams. With single electron beam energy included (6 MeV), they achieved total uncertainty within 2% for the calibration curve for dose levels above 0.8 Gy. Moylan et al.^(24)^ included 9 MeV electron beam in their study, where the EBT3 film was tested in *in vivo* dosimetry, concentrating on the film size, region of interest (ROI) size, and film scanning location dependencies. The combined dosimetric accuracy with the 6 MV photon beam was reported to be 2.6%. Farah et al.^(27)^ reported that the accuracy of their EBT3 film‐based system with the 6 MeV electron beam is comparable to the accuracy of the system with the 6 MV photon beam.

In this study, the dosimetric characteristics of the EBT3 film are investigated aiming to the applications of the EBT3 film for electron beam and combined photon/electron beam dosimetry. In addition to the fundamental dosimetry characteristics of the EBT3 film, the read‐out procedure is analyzed and some improvements to the procedure are presented. The study covers the electron beam energies 6 MeV, 9 MeV, 12 MeV, and 16 MeV, and presents energy dependence evaluation of the EBT3 film based on measurements and full Monte Carlo (MC) simulations. For the measurements of absorbed dose to water, uncertainty analysis in electron beams using the EBT3 film is presented.

## II. MATERIALS AND METHODS

### A. Radiochromic film, irradiation and scanning procedures

In this study, Gafchromic EBT3 film was used. It consists of two 0.125 mm thick layers of polyester foils and 0.030 mm active layer emulsion sandwiched between the polyester layers, the total thickness of the film being 0.280 mm. The surface of the film is covered with tiny silicone spheres eliminating Newton's rings artifact in the image. A more thorough description of the film structure is presented by Lewis et al.^(14)^ In this work, total of six boxes of EBT3 film (size $20\;\text{cm}\times 25\;\text{cm}\;(8^{\prime \prime }\times 10^{\prime \prime })$) from lots A12141101 (exp. Dec. 2013), 01171401 (exp. Jan. 2016), and 03031403 (exp. Mar. 2016) were used.

A single film sheet was cut to 5 cm×6 cm pieces for calibration irradiations and for irradiations to determine various film characteristics. For dose profile measurements, the film was cut to 5 cm×25 cm stripes, and dose distribution in plane was measured with a whole or a half film sheet, depending on the field arrangements and film orientation in the phantom. Identification marks were added on the edge of all films to record the original position and orientation. The films were handled in dimmed room light to minimize the potential unwanted background darkening of the film. The ${\;}^{60}\text{Co}$ gamma ray beam for the irradiations was from Gammabeam X200 (Best Theratronics Ltd., Ottawa, ON, Canada), and the photon and electron beams were from Varian Clinac iX (2300C/D) (Varian Medical Systems, Inc., Palo Alto, CA) linear accelerator (linac). Gammex 457 Solid Water (Gammex Inc., Middleton, WI) plates were used as phantom material for the measurements in the ${\;}^{60}\text{Co}$ gamma ray beam, whereas a water phantom was used in photon and electron beams. Apart from the films used to study the postirradiation darkening, the exposed films were scanned four days after the irradiation to ensure the stabilization of changes in all color channels. Films were scanned three times and an average value was determined. The background readings for each film pieces were collected no more than a week before irradiation and compared to average value of all film pieces. If the difference was more than 0.5% for red or green channel or more than 1% for blue channel, the film piece was rejected from the calibration.

All the films were scanned with Epson Perfection V750 Pro (Seiko Epson Corporation, Tokyo, Japan) flatbed scanner with an additional top lid for transmission images. Scanning was performed with 72 dpi resolution and 48‐bit color depth and no color corrections were used. The whole scanning area was utilized to maintain fixed X (transversal) and Y (longitudinal) positions in the image, the image size thus always being 576×720 pixels. All the images were saved in tagged image file format (tiff) for subsequent analysis. The film pieces were scanned with a film holder, which locates the film in the middle of the scanner plate and blocks the gaps for direct light from scanner. When film stripes were used, they were attached together with a tape for scanning to avoid small gaps between the films.

The films were analyzed with in‐house‐built Visual Basic .NET software (Microsoft, Redmond, WA). The program reads in 48‐bit tiff images from the scanner, analyzes the data from different color channels, and performs all required scanner corrections and dose conversions. The software is also able to combine several images to increase accuracy, and it can superimpose and compare the measured dose distributions to treatment planning system (TPS)‐generated dose distributions.

### B. Investigation of scanner‐dependent characteristics and corrections

The short term repeatability of the scanner was investigated by scanning the same unexposed and exposed films several times. Between each scan, 1 min delay was applied. The same procedure was repeated with a film exposed to 2 Gy dose. The long‐term repeatability was determined by scanning a single unexposed film, in which six ROIs and four position marks were added. The film was scanned 25 times over a period of four months. The pixel values (PVs) from ROIs and the distance between position marks were analyzed.

Correction for the inhomogeneity of the scanner sensitivity on the whole scanning area was determined by scanning 10 unexposed films and calculating the average value for each pixel and for each color channel. With this data, a relative correction matrix for selected normalization area was quantified. The normalization area, 3 cm×4 cm, was in the middle of the scanner plate and the same area was used for small films. The correction matrix was collected at least two times for each film lot. For unexposed film, this matrix correction produces uniform PVs for the whole scanning area.

In addition to the matrix correction, an additional dose dependent correction in transversal direction was required. This was determined by exposing a film stripe to a constant dose and scanning the stripe in different locations on the scanner plate. This additional dose dependent correction can be taken into account by applying an iterative method. The films stripes were exposed to a uniform dose with a rotating reel in a ${\;}^{60}\text{Co}$ gamma ray beam. The film stripe was attached inside the reel so that sufficient thickness of buildup and backscatter material was present on both sides of the film. The reel rotated over 400 rounds during irradiation, with uniformity less than 0.2% (1 SD) in the film. The dose was determined by comparing the irradiated films on the reel to the films calibrated in the calibration phantom.

### C. Description of dose determination

The net optical density (OD) for a film was determined as:

(1) $$OD_{net}={\text{log}}_{10}\left(\frac{PV_{un}}{PV_{ex}}\right)$$

where ${\text{PV}}_{\text{un}}$ is the PV for unexposed film and ${\text{PV}}_{\text{ex}}$ is the PV for the same exposed film. The background ${\text{PV}}_{\text{un}}$ was measured not more than a week before the irradiation.

The manufacturer of the film has provided guidance to fix the orientation of a film with respect to the scanner to avoid the errors in read‐out procedure due to film orientation.^(28)^ This effect was investigated comprehensively by measuring the film OD in steps of 10° rotation of the film. The effect of film orientation was taken into consideration as the procedure for read‐out of a film was improved through repeated read‐outs of film in different flipped orientations.

In this work, a combination of red and green channels was used for dose determination. A response of the EBT3 film to absorbed dose in water was investigated in ${\;}^{60}\text{Co}$ gamma ray beam to up to 8 Gy. The films were irradiated in the Gammex 457 Solid Water phantom at depth of 5 cm. The phantom block was placed in front of a 30 cm×30 cm30 cm PMMA wall water phantom to produce full backscatter. Source‐to‐skin distance (SSD) of 95 cm and field size 10 cm×10 cm were used, irradiating one film piece at a time, located in the center of the field. The calibration was done for each film lot at least three times. Typically three to five different dose levels ranging from 0.25 Gy to 8 Gy were given in each exposure, every calibration, including 2 Gy dose level. For ${\;}^{60}\text{Co}$ gamma ray beam reference doses were measured by a cylindrical Farmer‐type NE 2571 ($0.69\;{\text{cm}}^{3}$) ionization chamber (IC) (Nuclear Enterprises Ltd, Reading, England), connected to a NE Farmer dosimeter 2570/1. In all measurements with ICs in this study, IAEA TRS398 protocol^(29)^ was followed, and calibrations of ICs were traceable to standards of SSDL (STUK, Helsinki, Finland) and BIPM.

### D. Investigation of film‐dependent characteristics

#### D.1 Postexposure changes

The stabilization time for development of the EBT 3 film after irradiation was investigated with ${\;}^{60}\text{Co}$ gamma ray beam over a period of 16 days by scanning the same irradiated film several times in successive days. To investigate the influence of the scanner light, some of the films were scanned only few times during these 16 days.

#### D.2 Homogeneity

The homogeneity of film sheets was investigated as a part of the calibration procedure. The films were cut to 5 cm×6 cm pieces and the optical densities were measured from each 16 pieces prior to irradiation. More than 10 film sheets were used for calibration, resulting to that 160 film pieces in total were used. The measurement was done with all three color channels at 35 mm×40 mm area in the middle of each film piece.

#### D.3 Dose‐rate and energy dependence

The dose‐rate dependence was not subject to this study, but according to work by Casanova Borca et al.,^(21)^ the EBT3 film can be considered nearly independent on dose‐rate. The energy dependence of the dose response of the EBT3 film was investigated at reference measurement conditions by measurements and MC simulation. The measurements and simulations were made for 6 MV photon beam and 6 MeV, 9 MeV, 12 MeV, and 16 MeV electron beams of the linac. For each electron beam and 6 MV photon beam, at least two films were exposed to 2 Gy dose, minimizing the effect of dose dependence. All irradiations were made at the reference depth in a water phantom and the doses were measured with IBA PPC‐40 parallel plate IC (IBA Dosimetry AB, Sweden) and a cylindrical Farmer‐type NE 2571 IC connected to a NE Farmer dosimeter 2570/1. Sutherland and Rogers^(26)^ have pointed out the difficulties related to MC simulation of the EBT film dose response, emphasized especially at low‐energy photons. The consistency of simulated and measured responses of the EBT3 film with higher photon and electron energies was aimed for in our study.

The MC simulations were performed with the BEAMnrc code package (V4‐2.4.0, or BEAMnrc 2013), which uses the EGSnrc MC code system that simulates coupled electron–photon transport. The EGSnrc‐based phantom dose calculation is performed with DOSXYZnrc, which is also included in the BEAMnrc code package.^(30)^ The geometry model of the linac treatment head was based on the abovementioned linac applying proprietary manufacturer geometry and materials information. The MC model was based on the earlier work by one of the authors.^31^, ^32^, ^33^, ^34^ The iterative initial electron beam tuning process and beam parameter selection are discussed in Ojala et al.^31^, ^33^ The phase space data of the particles collected at the SSD 100 cm was used as a source in the dose calculation performed with the DOSXYZnrc code. The electron and photon transport cutoff parameters used were ECUT=AE=0.521 MeV and PCUT=AP=0.01 MeV. Other EGSnrc parameters were the same as in Ojala et al.^(34)^ In each DOSXYZnrc simulation, the number of particle histories used was selected so that the statistical uncertainty in high‐dose voxels was less than 0.3%.

To determine the absorbed‐dose energy dependence^(26)^ of the EBT3 film, DOSXYZnrc was used to simulate the dose deposition in the active layer of the film and corresponding volume replaced with water. A virtual rectilinear water phantom with 40 cm×40 cm area and 20 cm was modeled. For each electron beam energy, the film with exact materials and layer thicknesses was modeled at depth‐of‐dose maximum. For the photon beam, a depth of 5 cm was selected. The area of the scoring volume was 5 cm×5 cm. For the water used in the phantom, H20521ICRU was found in the default PEGS4 material library, but the polyester and the active material of the film were created by the authors with PEGS4 utility found in the BEAMnrc code package. The corresponding cross section data for the materials were applied in MC dose calculations. The phase space sources contained the particle data of a 20 cm×20 cm field for the electron beams and 10 cm×10 cm field for the photon beam. In MC simulation, the elemental compositions for materials of the EBT3 film shown in Table 1 were used.

**Table 1:** Elemental composition of the EBT3 film used in MC simulations.^(31)^

	*Composition (weight%)*				
*Layer*	*H*	*Li*	*C*	*O*	*Al*
Polyester	4.2	0.0	62.5	33.3	0.0
Active Layer	8.8	0.6	51.1	32.8	6.7
Polyester	4.2	0.0	62.5	33.3	0.0

## III. RESULTS

### A. Investigation of scanner‐dependent characteristics and corrections

Considering the short‐term repeatability of the scanner, the deviation with the unexposed film from average value was less than 0.2%, but with the exposed film the reading increased by 0.03% with each successive scan. Based on the long‐term repeatability measurements, the PV of a film can be determined within 0.2% accuracy.

The maximum deviation of PVs relative to longitudinal positions was 340 PV. The longitudinal deviations of the PVs are small compared to transversal direction and are not dose‐dependent. In transversal direction, the sensitivity does not depend notably on the longitudinal position on a scanner plate, whereas the sensitivity in transversal direction is asymmetric and in the right side of the image depends on the OD level (i.e., absorbed dose). With unexposed film, in transversal direction the matrix correction is in order of 300 PV on the left side of the image and decreases to zero in the middle and increases up to 1800 PV towards the right edge. Repeatability of the matrix correction is approximately 20 PV. To implement the additional dose‐dependent transversal correction an iterative procedure is followed. First, the correction for the background OD is made and an absorbed dose estimate for each pixel is calculated. In the second step, the dose estimates of the first round are used to calculate the dose‐dependent corrections for each pixel. For higher accuracy measurements, one can avoid the use of the right part of the image or the image can be scanned in four different orientations.

Corrected PVs relative to their position on scanner can be represented as follows:

(2) $$PV_{x,y,corr}=PV_{x,y}+M_{x,y}+MD_{x,dose}$$

where $PV_{x,y}$ is the raw measured PV, $M_{x,y}$ is the matrix correction, and ${\text{MD}}_{x,\text{dose}}$ is the dose‐dependent transversal correction. $PV_{x,y,corr}$ is converted to optical density according to Eq. (1).

**Figure 1:**
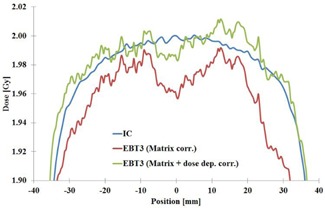
The verification of transversal correction of the film scanner. Measured relative dose profiles in ${\;}^{60}\text{Co}$ gamma ray beam for 10 cm×10 cm field at depth of 5 cm in a phantom. Blue=IC measurement,Red=EBT3 measurement with the matrix correction, and Green=EBT3 measurement with the matrix correction and dose‐dependent correction.

Transversal dose‐dependent correction was verified by comparison to ${\;}^{60}\text{Co}$ gamma ray beam absorbed dose profiles measured in water by Scanditronix‐Wellhöfer RK cylindrical IC (Scanditronix Medical AB, Uppsala, Sweden) and the EBT3 film. The experiment was made at 2 Gy dose level. The measured profiles are shown in Fig. 1. Inclusion of dose‐dependent transversal correction produces dose profile comparable to measurement by IC within 0.5%.

### B. Description of dose determination

**Figure 2:**
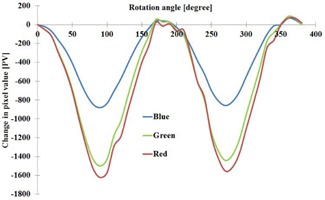
The relative change of OD in the middle of a film sheet in a rotation point as a function of the angle of rotation. The maximum difference is with 90° and 270°. In zero angle position the longer edge of the film sheet is parallel to scanning direction. OD value is a mean OD in a circular area of 30 mm in diameter. Blue=blue channel measurement,Green=green channel measurement, and Red=red channel measurement.

The variation of OD relative to rotation of the film on scanner plate is presented in Fig. 2. Sinusoidal type variation of OD was received at 90° intervals of rotation. The largest change occurred at 90° rotation, but with 0° and 180° the results remained constant. The films can be scanned either in landscape or in portrait orientation, but this has to be fixed for all scans. However the film can be rotated 180° and flipped around with no change of net OD.

**Figure 3:**
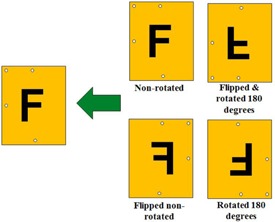
Procedure for scanning the EBT3 film image in four different orientations. Only 180° of rotation is made.

As described in by Lewis et al.,^(14)^ the EBT3 film is symmetrical and can be read on both sides. The variation of OD relative to the angle of rotation was found to be identical within the uncertainty related to the repeatability, if the measurement is performed on either side of the film. Exploiting this feature of the EBT3 film, a following enhanced read‐out procedure based on flipping the film sheet was established (see Fig. 3).

The film sheets are marked for identification, orientation, and superimposition. The film is scanned in four different orientations; non‐rotated, rotated 180°, flipped non‐rotated, and flipped and rotated 180°. All four images are corrected with the scanner corrections and converted to dose. Then the dose distributions are rotated, mirrored, and superimposed to restore the original orientation and images are merged by mean dose.

**Figure 4:**
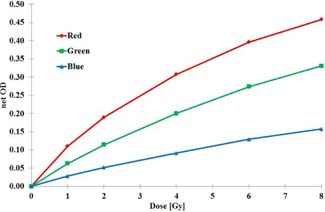
The net OD values for all color channels as a function of the absorbed dose to water in ${\;}^{60}\text{Co}$ gamma ray beam (dose rate 1.4 Gy/min) with dose levels up to 8 Gy. A linear fit has been applied between data points. Film lot: 01171401 (exp. Jan. 2016). Red=red channel measurement,Green=green channel measurement, and Blue=blue channel measurement.

The measured net OD values as a function of absorbed dose to water is presented in Fig. 4. To determine the absorbed dose from the net OD values, a following function was found to produce the best fit to the experimental data for all color channels:

(3) $$D=\frac{a+cx}{1+bx}$$

where *x* is the net OD. The negative OD values must be handled accordingly to set the zero level for dose properly and extrapolation for higher doses should be avoided. Also, the dose response and the fitting parameters are expected to depend on the film lot used, on the variation between individual scanners and changes caused by aging of the film.

Based on the measured and fitted data, an OD‐dependent calibration factor for the EBT3 film is defined as:

(4) $$N_{W,Q,OD}=\frac{D_{W,Q}}{OD_{M,Q}}$$

where the *Q* refers to radiation quality used in calibration.

### C. Investigation of film‐dependent characteristics

#### C.1 Postexposure changes

**Figure 5:**
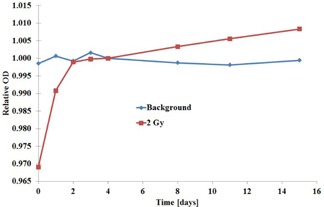
Relative OD as a function of time from exposure to read‐out. Irradiation with ${\;}^{60}\text{Co}$ gamma ray beam. Time in days and OD normalized to OD value at four days. Blue=background (unexposed EBT3 film) and Red=exposed to 2 Gy absorbed dose.

After relatively fast increase in OD during first two days, the increase in OD becomes slower. For a period from four to 16 days, the increase in OD was about 1% (Fig. 5). For practical reasons, a four‐day delay from irradiation to read‐out was fixed for other measurements. In the time span from two to four days, the change in OD is less than 0.2%.

#### C.2 Homogeneity

The background determination was done for 160 film pieces and the maximum variations of ODs for all channels were within 2%. For all 50 film pieces used in dose calibration, the maximum variations of ODs for both red and green channels were within 0.5% and for blue channel within 1%. The blue channel response is affected the most on the thickness variation of the film emulsion. If the response of a single film piece deviated more than 1% in blue channel response from the average value, the film piece was not used for calibration. Only a few pieces had to be rejected from the calibration, which implies that the overall homogeneity of the films was very good. Maximum difference of these rejected films was 2%. This analysis is based, however, on only three different lots and six packages of film. Each film package should be verified separately. Based on the experience in this study, the OD of a film sheet varies in a shape of longitudinal bands, which are assumed to be due to spreading of emulsion in the film. For this reason when stripes of film are used, it is recommended to cut the film in longitudinal direction.

#### C.3 Energy dependence

**Figure 6:**
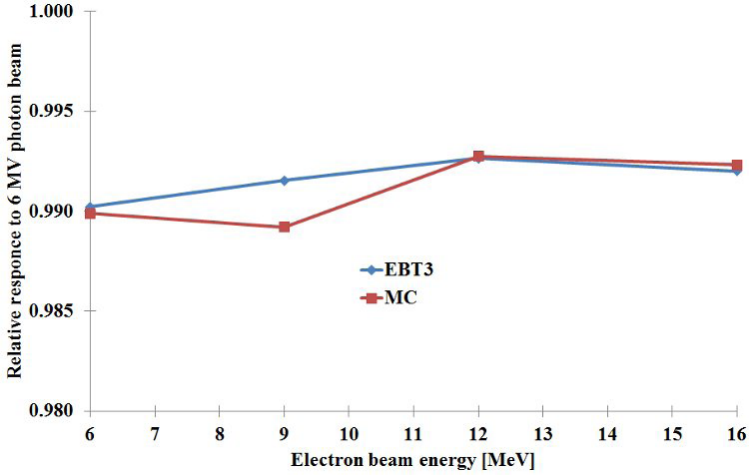
The response of the EBT3 film for absorbed dose to water as a function of electron beam nominal energy and normalized to the response for 6 MV photon beam. Blue=EBT3 measurement and Red=MC simulation. The uncertainty of measured absolute dose for 6 MV photon beam is 1.3% (k=1) and the main contribution of uncertainty comes from $k_{Q}$ factor of the IC. The contribution to the uncertainty from the film measurement is 0.5% (k=1). The statistical uncertainty of MC simulations was within 0.3%.

The MC‐simulated and measured energy dependencies of the EBT3 response at reference measurement conditions for 6 MV photon beam and 6 MeV, 9 MeV, 12 MeV, and 16 MeV electron beams are presented in Fig. 6. The reference measurement depths for 6 MV photon beam and 6 MeV, 9 MeV, 12 MeV, and 16 MeV electron beams were 50 mm, 13 mm, 20 mm, 29 mm, and 38 mm, respectively. As the results suggest, when normalized to the ratio of the value for the water and the EBT3 film for the 6 MV photon beam, all the results for the electron beams are within 0.5%. Also, the difference between the experimental and simulated responses is negligible, indicating minimal intrinsic energy dependence of the EBT3 film at this energy range. The difference in the EBT3 response between 6 MV photon beam and electron beams from 6 MeV to 16 MeV is about 1.0%.

### D. Uncertainty budget

The uncertainty evaluation is presented in Table 2 for a situation where the EBT3 film is calibrated in a 6 MV photon beam, and measurements are performed in electron beam and where all measurements are performed in water filled PMMA phantom. For Step 1, the largest contribution to uncertainty comes from implementing the beam quality correction factor of the IC from ${\;}^{60}\text{Co}$ gamma ray beam to 6 MV photon beam, according to TRS‐398 protocol.^(29)^ In Step 2, ±0.5 mm accuracy in positioning of the film in a water phantom was assumed, which can be regarded as conservative estimate for reference conditions. This leads to 0.58% (1 SD) uncertainty in dose, which is the largest sub‐uncertainty in Step 2. For Step 2, the relatively low total uncertainty 0.71% (k=1) was achieved due to low statistical uncertainty and minimal corrections for uniformity for scanner on 35 mm×40 mm area. In actual measurement of electron dose distribution at linac beam (Step 3), the main components for the uncertainty are measurement of OD on large area film (0.62%, 1 SD) and the positioning of the film in a water phantom (0.58%, 1 SD). For the measurement of relative dose distributions in electron beams uncertainty of 2.3% (k=2) was concluded. This included the uncertainty of Step 3 shown in Table 2, added with uncertainty contribution of normalization of film result to corresponding area of the IC.

**Table 2:** The uncertainty budget for the EBT3 film. Absorbed dose level close to 2 Gy corresponding 26000‐26500 PVs. The film calibration in 6 MV photon beam and measurement of electron dose distribution. Resolution 72 dpi.

	*Uncertainty* (k=1,%)	
*Source of Uncertainty*	*For the Step (1 SD) %*	*Cumulative (1 SD) %*
Step 1. Measurement of absorbed dose to water with IC at SSD 100 cm, 10 cm×10 cm field at 10 cm depth in water phantom in 6 MV photon beam.	1.40	–
Step 2. Calibration of film (OD on area of 35 mm×40 cm) at SSD 100 cm, 10 cm×10 cm field at 10 cm depth in water phantom in 6 MV photon beam.	0.71	1.57
Step 3. Measurement of absorbed dose to water by the EBT3 film in electron beam. Single PVs on 200 mm×250 mm scanning area.	1.00	1.86
Combined expanded uncertainty (k=2)	–	3.72

## IV. DISCUSSION

### A. Investigation of scanner‐dependent characteristics and corrections

The short‐term repeatability of the EPSON Perfection V750 Pro flatbed scanner can be considered suitable for the EBT3 film dosimetry. The unexposed film is quite insensitive for scanner light, but the exposed film showed some sensitivity. If five or less scans are taken for each film, a mean value can be used for reading. If more scans are repeated for each film, it is recommended to calculate a trend line and interpolate the reading to the first one. All measurements were done successfully with 72 dpi reading accuracy (i.e., 0.3 mm×0.3 mm pixel size), which implies that the spatial resolution of the EBT3 film dosimetry system is sufficient for clinical treatment plan verification purposes. From practical point of view, it was noticed that some films are slightly bent and a holder was needed to press the corners of the film towards the scanner plate.

The inhomogeneity of the scanner sensitivity needed to be corrected to achieve reliable results for measured dose distributions. Different methods have been presented for corrections by other groups.^35^, ^36^ By the two‐phase corrections implemented in this work, the homogeneity of scanner response can be improved remarkably. However, large area scanners would be beneficial for film scanning, as the uniform scanning area is comparable to EBT3 film size.^(35)^ The applied corrections for scanner inhomogeneity account for the largest uncertainty component of 0.62% (1 SD) for measurement of absorbed dose distributions. It is assumed that the main cause of the inhomogeneous response is due to complicated optic system with mirrors used in the plane scanners. It is obvious that the changes in scanner sensitivity are dependent on the scanner used and should be determined for each scanner individually.

### B. Description of dose determination

By flipping the film for scanning, the uncertainties related to scanner corrections are smoothed and precision is improved by repeated readouts. The method reveals also easily, if the homogeneity corrections of the scanner are not adjusted properly. The random errors in PVs related to dust on film can be detected and corrected before any smoothing. However, the elimination of effects of scratches or permanent dirt on film requires more comprehensive analysis of image data. In this work, the responses from red and green channels were averaged, which decreased the effect of noise present in values from separate channels. It was observed that the use of green and red channels is advantageous, since they are more sensitive than the blue channel. However, the response from blue channel can still be used when calculating average values in larger areas — for example, background film evaluations.

It was noted that to achieve accuracy level of 2%–4%, each new film lot needs an initial and repeated calibrations. Our work confirmed the recommendations by Dreindl et al.^(13)^ that recalibration should be performed every three months. The accuracy can be improved, if the calibration of the EBT3 film is performed at the same time as the actual measurement. This minimizes the effect of scanner long term repeatability, background level of film lot, and possible environmental effects of the film storage. For absolute electron beam dose measurements, the calibration of the EBT3 can be made in ${\;}^{60}\text{Co}$ gamma ray beam, in megavoltage photon beam or in megavoltage electron beam. The lowest uncertainty can be achieved if the film is calibrated in 6 MV photon or ${\;}^{60}\text{Co}$ gamma ray beams (3.7%,k=2). Close to that uncertainty is achieved, if cylindrical IC is used as reference in electron beam and clearly lower accuracy is achieved, when plane parallel IC is used. In all the IC measurements, IAEA TRS‐398 or other recognized international dosimetry protocol is recommended to be used. In the calibrations in this study, in most cases the film was irradiated to 2 Gy dose and the reference film was irradiated to same dose in order to minimize the nonlinear calibration of the film. A preliminary measurement of electron beam energy dependence was made in Gammex 457 Solid Water phantom and a 3% difference was noted between MC calculations and measurements with lower electron beam energies. With further investigations, the Solid Water was found nonwater‐equivalent, with electron beam energies lower than 12 MeV. All measurements after that were made in water phantom and excellent agreement was achieved between measurements and MC calculations.

### C. Investigation of film‐dependent characteristics

#### C.1 Postexposure changes

The period from irradiation to read‐out of the film was investigated and 96 h was concluded to be most practical for external dosimetry audits. Between two to four days the change in sensitivity is 0.2%. Four‐day pre‐read period with ±12 h time tolerance for the read‐out time was concluded to provide reliable results and to help read‐out also in specific cases. Four‐day pre‐read period allows travel or other delays between the irradiation and read‐out. Slightly lower uncertainty can be achieved if the read‐out would be performed in less than ±6 h time tolerance for the read‐out time. Shorter, down to 48 h pre‐read period can be used with level of 0.4% maximum deviation, if ±0.5−1 h time tolerance for the read‐out time is followed.

#### C.2 Homogeneity

The EBT3 film structure is symmetrical and double plastic layers give good rigid form for the film. Based on our experience, the maximum variation in sensitivity of a film sheet is less than 2%. This is clearly lower value than for the EBT2 film, where up to 5% variations were noticed by the authors (based on unpublished results). Some band‐shaped sensitivity changes, indicated by measurement with the blue channel, were noticed in the EBT3 films. Blue channel can be used to control the homogeneity of individual film sheets according to tolerances in use.

#### C.3 Energy dependence

The energy dependence of the dose response of the EBT3 film was investigated by measurements and by MC simulation. In 6 MV photon and electron beams ranging from 6 MeV to 16 MeV and at reference measurement conditions, dose response of the EBT3 film was found to be uniform within 1.0%, with uncertainties close to 1.58% (k=2). Change in response from ${\;}^{60}\text{Co}$ gamma ray beam to 6 MV photon beam is negligible. The nearly uniform energy dependence implies that the EBT3 film can be used for measurements of dose distributions in mixed photon and electron beams and in measurements where radiation beam quality correction cannot be made, with reasonably low contribution of uncertainty due to the energy dependence. These results are strictly valid for reference conditions and electron energy spectra present at reference measurement depth. For further studies, investigation of electron beam responses at larger depths should be evaluated. Inaccuracies related to dose dependence of the EBT3 film response can be minimized, when similar dose levels are used in calibration and actual measurements, when using the EBT3 film.

Sutherland and Rogers^(26)^ have pointed out the difficulties related to MC simulation of the EBT film dose response, especially at low‐energy photons. They concluded that contribution of intrinsic energy dependence (ratio of dose to the sensitive volume and detector reading) cannot be simulated by MC methods due to possible changes in emulsion polymerization process of EBT film relative to photon energy. They considered this as a possible reason for deviation of measured and calculated results at low‐energy photons. They also concluded that other source causing differences between the published measured and MC‐calculated results could be the differences of film sheets in different film lots. In our study, no noticeable difference was found between the experimental and simulated responses, indicating negligible change in intrinsic energy dependence of the EBT3 film for 6 MV photon beam and 6 MeV–16 MeV electron beams at reference measurement conditions.

### D. Uncertainty budget

The main contribution of uncertainty for measured absorbed dose by the EBT3 film comes from inhomogeneity of the scanner sensitivity and the related corrections, especially in the dose‐dependent correction in transversal direction. In addition to uncertainty of measured OD, the notable components of uncertainty in absolute dose measurement in electron beam originate from the measured reference dose by IC and positioning of the film in a phantom. The film could be calibrated in 6 MV photon beam, and used for measurements of electron or mixed photon and electron and photon dose distributions within uncertainty of about 3.7% (k=2). For measurements of relative dose distributions, uncertainty of 2.3% (k=2) is expected.

Compared to measurement of optical sensitivity in single pixels, scoring dose in larger area, such as 35 mm×40 mm, reduces the uncertainty notably. Uncertainty of OD for single pixel is 0.7% (1 SD) on scan area of 20 cm×24 cm (with 72 dpi) and 0.4% (1 SD) for average dose on area of 35 mm×40 mm in the middle of the scan area of the scanner. These results are consistent with the results by Sorriaux et al.,^(10)^ where uncertainty of 0.55% (1 SD) was concluded for optical density. Uncertainty estimate assumes close to 2 Gy dose level and higher uncertainties of OD for lower doses were imminent for scanner used in this study, because the same uncertainty in PV was obtained independently of PV level.

## V. CONCLUSIONS

Based on the dosimetric characteristics the EBT3 film, the read‐out procedure established and the estimated uncertainties, the EBT3 film was concluded to be a suitable 2D dosimeter for measuring electron or mixed photon/electron dose distributions in a water phantom.

## ACKNOWLEDGMENTS

This work is part of the European Metrology Research Programme Joint Projects, HLT09 MetrExtRT, “Metrology for radiotherapy using complex radiation fields”. The authors declare that they have no conflicts of interest.
